# Review on Prescription Compatibility of Shaoyao Gancao Decoction and Reflection on Pharmacokinetic Compatibility Mechanism of Traditional Chinese Medicine Prescription Based on *In Vivo* Drug Interaction of Main Efficacious Components

**DOI:** 10.1155/2014/208129

**Published:** 2014-07-23

**Authors:** Xiaolin Bi, Meirong Gong, Liuqing Di

**Affiliations:** ^1^College of Pharmacy, Nanjing University of Chinese Medicine, 138 Xianlin Avenue, Nanjing Jiangsu 210023, China; ^2^Jiangsu Engineering Research Center for Efficient Delivery System of TCM, Nanjing 210023, China; ^3^Nanjing Engineering Research Center for Industrialization of Chinese Medicine Pellets, Nanjing 210023, China; ^4^The Second Clinical Medical College, Nanjing University of Chinese Medicine, Nanjing 210023, China

## Abstract

Shaoyao Gancao Decoction (SGD) derived from Zhang Zhongjing's “Typhoid Theory” is composed of peony and licorice, having the efficacy of nourishing liver, relaxing spasm, and relieving pain. Modern compatibility studies of SGD on chemistry, pharmacology, and pharmacokinetics all demonstrate the reasonable compatibility of peony and licorice. However, the present research on pharmacokinetics is only descriptive and limited to the influence on *in vivo* dynamic process of certain ingredients; correspondingly, there is lack of studies on the essence of these efficacious substances' *in vivo* changes; that is, whether it is because there exists *in vivo* drug interaction in absorption, distribution, metabolism, and excretion (ADME) of active ingredients that leads to the improvement of bioavailability. We herein take SGD as an example and suggest that it is necessary to study *in vivo* drug interaction of main efficacious components mediated by metabolic enzymes, transport proteins, or plasma protein binding in the course of ADME, which is helpful to illustrate the principle of pharmacokinetic compatibility from the essence leading to the changes of effective substances *in vivo*.

## 1. Introduction

Formulae are the main clinical patterns of Chinese medicine and the core of formulae is prescription compatibility. Thus prescription compatibility becomes a critical issue in prescription study. Research on compatibility principles and mechanisms of “monarch, minister, assistant, and messenger” and “seven herbal emotions” is of great importance in revealing the myth of prescription compatibility and directing modern research and development on components compatibility of herbal medicine. In the development of herbal modernization in China, modern studies of prescription compatibility have witnessed great advances all the way from material crude slices to ingredients,* in vitro* to* in vivo*, and single method to multiple scientific methods [1].

Shaoyao Gancao Decoction (SGD) derived from Zhang Zhongjing's “Typhoid Theory” is composed of peony and licorice, having the efficacy of nourishing liver, relaxing spasm, and relieving pain. The content of paeoniflorin is the highest among all effective components in peony, which has the effect of spasmolysis, analgesia, sedation, cool antipyretic, and antioxidation [2]. The main active ingredients of licorice can be divided into three major categories: three terpenoids (glycyrrhizin, glycyrrhetate, and glycyrrhetinic acid), flavonoids (licorice flavanone, etc.), and* Glycyrrhiza* polysaccharide, having the function analogous to the adrenal cortical hormone and the functions of antiulcer, antispasm, anti-inflammation, antiallergy, antivirus, detoxification, cough expectorant, and antitumor [3]. This paper reviews the modern compatibility studies of SGD in terms of chemistry, pharmacology, and pharmacokinetics and proposes to investigate pharmacokinetic compatibility mechanism on the basis of* in vivo* drug interaction of main efficacious components, which would be helpful for finding the compatibility principles from the essence of* in vivo* changes of the active substances and would also be an important way and means to explain the mechanism of traditional Chinese medicine prescription compatibility.

## 2. Modern Studies of SGD Compatibility

### 2.1. Chemical Aspect

The changes of material basis before and after prescription compatibility of SGD are embodied in two aspects: the* in vitro* changes are mainly presented in cooking, and the* in vivo* changes are manifested in ADME. Qiu and coworkers [4] investigated the influence on the quantity of paeoniflorin when peony and licorice were decocted in a ratio of 1 : 1, 1 : 2, and 2 : 1, respectively, and discovered that different dosage proportions had no impact on the content of paeoniflorin. Tan et al. [5] concluded that there were no chemical changes before and after prescription compatibility by comparing the HPLC-UV fingerprints of individual decoction and admixture decoction of SGD. However, Shen et al. [6] found seven new ingredients by adopting HPLC-DAD-ESI-MS in terms of the* in vitro* changes of chemical composition, but found eight new ingredients in serum of rats administrated with SGD. Chen [7] compared HPLC fingerprints and medicated serum HPLC fingerprints between single herb decoction and Chinese herb medicine compound decoction. Their results demonstrated that there were eight components from peony, twenty-three components from licorice, and thirty-two components from SGD in serum, respectively; some peak areas of serum samples were obviously increased, including albiflorin (SGT6), paeoniflorin (SGT7), liquiritin (SGT11), glycyrrhizin (SGT29), and glycyrrhetinic acid (SGT32). They concluded that the bioavailability was significantly improved after the combination of peony with licorice, which showed synergistic effects between them. Shen et al. [8] administrated the rats with the formula composed of three active compositions of paeoniflorin, glycyrrhizic flavones, and glycyrrhizic acid, and the obtained serum HPLC fingerprints showed some new active metabolites, which had high correlation with efficacy.

### 2.2. Pharmacological Aspect

Xu and Jin [9] investigated the effect of different proportions of SGD on primary dysmenorrhea and found that SGD could reduce the writhing time of the model rats caused by pitocin, and the 3 : 1 group achieved the best efficacy. By using xylene to induce auricular swelling in mice, tampon to make rats granuloma, egg white to make pedal swelling in rats, and hot plate and acetic acid to make body torsion in mice, Liu and coworkers [10] found that the total glucosides from SGD had anti-inflammatory and analgesic actions, in which the total glucosides from* Glycyrrhiza uralensis *and the total glucosides from* Paeoniae Alba* had synergistic action. Liu et al. [11] studied the inhibition and compatibility of SGD on phospholipase A2 (PLA2) by fluorescent liposome and found that SGD had a greater inhibitory effect than single* Glycyrrhiza uralensis* and single* Paeoniae Alba.* The effective component clusters of SGD were composed of paeoniflorin, glycyrrhizic acid, and licorice flavonoids in Gu's research [12], and the analgesia effect was the best when the ratio was 1 : 1 : 2. Zhang et al. [13] investigated the synergistic potential of total glucosides of Paeoniae Radix (TGP) and total flavonoids of Glycyrrhizae Radix (TFL). Isobolographic analysis revealed that the combination of TGP and TFL in the fixed ratio of 3 : 1 exerted the highest synergistic interaction. SGD could also downregulate Sirt1 protein expression, which was 4.2-fold higher than that of model rats in dorsal root ganglion.

### 2.3. Pharmacokinetics Aspect

#### 2.3.1. The Influence of Compatibility of Peony and Licorice on Pharmacokinetic Properties of Active Ingredients

Wang et al. [14] compared the pharmacokinetic parameters of paeoniflorin and glycyrrhetinic acid in serum of SD rats administrated with peony, licorice, and SGD, respectively. The results showed that compared with the sole administration with peony or licorice, glycyrrhetinic acid in serum from SGD reached the peak much faster with an increased Cmax; paeoniflorin's peak value was enhanced, and so did the relative bioavailability; and the ingredients both showed reduced half-life. Rats were orally administered with SGD, Shaoyao decoction and Gancao decoction, respectively, and the compatibility of the SGD was revealed in terms of pharmacokinetic characteristics in Shen and coworkers' research [15]. They found that the absorption of Shaoyao components was increased in SGD, while the absorption of Gancao components was time-dependent. In SGD, the increased absorption of some Shaoyao components may be related to a reduction in absorption of some Gancao components. By comparing the pharmacokinetic parameters and bioavailability of glycyrrhizic acid and glycyrrhetinic acid after oral administration of licorice decoction and SGD in rats, Xiang et al. [16] found that both blood concentration and bioavailability of glycyrrhizic acid and glycyrrhetinic acid in SGD group were significantly increased, indicating the synergism in the action of SGD. After the effective combination of paeoniflorin (44% purity), glycyrrhizic acid (50% purity), and liquorice flavones (52% purity), glycyrrhizic acid (50% purity) and liquorice flavones (52% purity) were administered to rats, respectively, and Shen et al. [17] found that effective combination group had higher AUC and Cmax and longer *T*_max⁡_ and slower clearance rate in rat blood than glycyrrhizic acid group and liquorice flavones group, demonstrating the advantage of the effective combination of* Paeonia lactiflora* and* Glycyrrhiza uralensis*. Hu et al. [18] studied the pharmacokinetic changes of the characteristic ingredients in the combination of different-dose herbs of SGD and found that the best dose ratio was 4 : 4, which was consistent with the dose commonly used in ancient times. The absorption of characteristic peaks from SGD was related with the dosage of Gancao, and there existed interaction between each characteristic ingredient. The compatibility connotation in a right dose-ratio of SGD has been demonstrated in terms of pharmacokinetics. Xu et al. [19, 20] compared pharmacokinetics of ten bioactive compounds in rat plasma after oral administration of different combinations (*Paeonia* :* Glycyrrhiza*, 1 : 1 or 1 : 4) of SGD and found that there were perceptible differences in pharmacokinetic parameters (Cmax, AUC_0-*t*_, and CL) of the analytes except for liquiritin between the two groups. Albiflorin and paeoniflorin are the main effective compounds of* Radix Paeoniae alba*, and their pharmacokinetic differences in rats after oral administration of Shaoyao-Gancao-Tang (SGT) and single herb peony decoction were studied in Gan and coworkers' research [21]. The results indicated that some components in the other ingredient herbs of SGT (*Radix Glycyrrhizae*) had pharmacokinetic interaction with albiflorin and paeoniflorin and hence reduced their systematic exposure level.

Considering the fact that composition of Chinese medicine prescription is complex, the quality is hard to control, and the therapeutic mechanism is difficult to explore; some scholars have only studied the interactions between effective monomer components. For example, Li and coworkers [22] found that glycyrrhizin significantly influenced the pharmacokinetic fate of paeoniflorin by increasing the value of AUC and decreasing CL and Vd when paeoniflorin and glycyrrhizin were administrated to rats via vena caudalis. Sun et al. [23] found that when glycyrrhizic acid was coadministered with paeoniflorin, the *ρ*_max⁡_ and AUC of glycyrrhizic acid were decreased to 9% and 33% of that of glycyrrhizic acid administered alone, respectively; the *T*_max⁡_ prolonged markedly; CL increased; and Vd became larger. For glycyrrhetinic acid, only the *t*_1/2_ was prolonged significantly, while other PK parameters had little changes. The results showed that paeoniflorin dramatically inhibited the absorption rate and extent of glycyrrhizic acid in rats when simultaneously orally administered but had only small effect on the absorption of glycyrrhetinic acid.

#### 2.3.2. The Influence of Compatibility of Peony and Licorice on Absorption Properties of Active Ingredients

Li et al. [24] studied the effect of glycyrrhizin on intestinal absorption of paeoniflorin using the everted gut sac model in rats and found that glycyrrhizin could reduce the uptake of paeoniflorin, which was maybe associated with the improved efflux from intestinal cells or enhanced biotransformation of paeoniflorin. Xin [25] investigated the intestinal absorption of main components and extracts compatibility of Shaoyao and Gancao. The results showed that, compared to monomer, the absorption percentage of the glycyrrhizin acid was decreased by 3.3% in duodenal, 6.25% in jejunum, 13.08% in ileum, and 3.18% in colon, respectively, and the paeoniflorin was decreased by 12.93% in ileum when glycyrrhizin acid and paeoniflorin were administrated to the rats in the ratio of 1 : 1; the absorption percentage of the glycyrrhizin acid was increased a little by 9.53% and 4.59% in the ileum, respectively, and the absorption percentage of the paeoniflorin decreased a little by 12.94% and 11.32% in the ileum, respectively, when glycyrrhizin acid and paeoniflorin were administrated to the rats in the ratio of 1 : 5 or 5 : 1; compared to monomer, absorption percentage of paeoniflorin was decreased by 6.02% in ileum when liquiritin and paeoniflorin were administrated to the rats in the ratio of 1 : 1. However, compared to single extracts of* Paeoniae Radix*, the absorption percentage of paeoniflorin was increased by 6.02% in duodenum when the extracts of Shaoyao and Gancao were administrated to the rats in the ratio of 1 : 1; compared to single extracts of* Glycyrrhiza uralensis*, the absorption percentage of glycyrrhizin acid was increased by 11.34% in duodenum, 9.39% in jejunum, 10.71% in ileum, and 6.63% in colon, respectively, when the extracts of Shaoyao and Gancao were administrated to the rats in the ratio of 1 : 5, but there was no significant difference after statistical analysis.

Paeoniflorin was the substrate of P-glycoprotein (P-gp) [26]. Glycyrrhizin and glycyrrhetinic acid could affect the function and expression of P-gp in Caco-2 cell monolayer [27] and inhibit the efflux mediated by P-gp [28, 29], which could be partly attributed to the inhibitory effect on ATP activities by glycyrrhetinic acid. In addition, glycyrrhizin and glycyrrhetinic acid might also be affected by P-gp when absorbed. It is postulated that absorption changes of SGD compatibility are related with the interaction mediated by P-gp.

#### 2.3.3. The Influence of Compatibility of Peony and Licorice on Metabolism Properties of Active Ingredients

The metabolism of paeoniflorin may also account for its low bioavailability. Studies showed that most glycosides disappeared in the upper small intestine, which were hydrolyzed into aglycons first and then absorbed in the form of aglycons whose permeation speed was 48 times higher than glycosides [30].* Glycyrrhiza uralensis* and its main component glycyrrhizin had influence on the mRNA expression of CYP1 and CYP2 isozymes and may change the activity of CYPA3 [31–34]. Glycyrrhetinic acid had influence on CYP1 and CYP2 activity [31, 35]. Liquiritin could regulate the CYP3A4 activity [36]. CYP1A2 was one of the enzymes involved in metabolism of isoliquiritigenin [37], indicating that isoliquiritigenin was probably the substrate of CYP1A2. In addition, 18*α*-glycyrrhizic acid could inhibit the activities of CYP3A4, CYP2E1, and CYP1A1 and induce the activities of phase II enzymes (GT1, GT2, and GST) [38]. The inductive effect on CYP metabolic enzymes could explain why* Glycyrrhiza uralensis* could relieve the drug toxicity.

#### 2.3.4. The Influence of Compatibility of Peony and Licorice on Plasma Protein Binding of Active Ingredients

The binding rate of paeoniflorin and albiflorin to human or rat plasma protein was relatively high, and the binding rate was identically proportional to their corresponding plasma concentration in liu' research [39]. Chen and colleagues [40] determined 18H-glycyrrhizin (18*α*-GL and 18*β*-GL) and 18H-glycyrrhetinic acid (18*α*-GA and 18*β*-GA) concentration in human plasma by GC and calculated plasma protein binding rate. The results demonstrated that when the human plasma was less than 50%, the binding rate of 18*α*-GL with plasma protein was higher than that of 18*β*-GL; while the concentration of the human plasma was over 50%, the result was reversed. The binding rate of 18*α*-GA was lower than that of 18*β*-GA all the time.

### 2.4. Conclusion

All the studies discussed above indicate the reasonable compatibility of peony and licorice in term of the* in vivo* changes in both quantity and quality of chemical ingredients, pharmacodynamics, and pharmacokinetics, suggesting that there is drug interaction when peony is coadministered with licorice, which affects the ADME of active ingredients.

However, the present research on pharmacokinetics is only descriptive and limited to the influence on* in vivo* dynamic process of certain ingredients. It just gives reasonable interpretation of formulae compatibility from phenomena, with little research on link and mechanism of pharmacokinetic compatibility; that is, there is lack of research on the root causes that leads to the changes of pharmacodynamic substances* in vivo*. The descriptive evidence is not enough for recognizing the nature of pharmacokinetic compatibility.

## 3. Reflection on Pharmacokinetic Compatibility Mechanism of Traditional Chinese Medicine Prescription Based on* In Vivo* Drug Interaction of Main Efficacious Components

### 3.1. Understanding of* In Vivo* Drug Interaction of Traditional Chinese Medicine Prescription Compatibility

Drug interaction usually refers to the quantified changes of certain medicine owing to the administration of other medicines before or at the same, which then exhibits the efficacy or toxicity changes. The formula made of several herbs is such a huge chemical compound library that the pharmacological actions on the organism must be comprehensive regulation by multiple chemical compositions. Therefore, the pharmacokinetic interaction might be the same as the effect while combined with western medicine. On the one hand, the extract coming from “minister, assistant, and guide” can be the basic active substances combined with the ingredients coming from the “monarch” to exhibit multiple targeted actions. From the view of pharmacokinetics, the effective ingredients from “minister, assistant, and guide” will influence the absorption, distribution, and metabolism of those from “monarch.” On the other hand, the technology system concerning “comparable evaluation of plant medicines” in international medical circles reveals the following: plant extracts can be divided into basic active substances and other accompanying substances. The accompanying substances can change the physicochemical properties of basic active substances and then have effect on biopharmaceutical characteristics, especially in the dissolution and further absorption of active substances from the prescriptions or the extracts [41].

Pharmacokinetic interaction of the herb covers ADME. The absorption of oral drug is affected by physicochemical properties of the drug itself, such as solubility, oil-water partition coefficient, and stability. It is also affected via intestinal transporters, drug metabolic enzymes (intestine has many types of phase I metabolic enzymes such as cytochrome P450 (CYP450), hydrolytic enzyme, and dehydrogenase and phase II metabolic enzymes such as UDP-glucuronyl transferase (UDPGT), sulfate transferase (ST), methyltransferase (MT), and intestinal flora metabolism (mainly hydrolytic reaction and reduction reaction)). The transport proteins having fully been studied so far are MDR1 (P-gp), BCR, MRP2, and so forth. Among them, P-gp can transport the drug from basilar membrane of gastrointestinal epithelium to top-lateral membrane, so as to pump it outside the cell, which hinders drug absorption and decreases oral bioavailability of some drugs. The substrates, inducers, and inhibitors of P-gp generally exist in the common drugs, so the drug transport mediated by P-gp is an important mechanism leading to the pharmacokinetic interaction [42]. The metabolic enzyme of many drugs can be induced or inhibited by other drugs administrated simultaneously, which is termed metabolic interaction, and the result is that the concentration of substrates or metabolites in blood or tissue is remarkably decreased or improved, or the toxicity is accumulated, which may change the efficacy and safety of drugs, especially those having narrow curative window. Many studies show that enzymes and transport proteins in intestine and liver mediate absorption and metabolism of drugs and lead to a series of interactions among drugs. It is known that the drug can arrive at the targeted location only after going through several biological membranes. However, the P-gp distributed on barriers such as blood-brain barrier, blood-testicle barrier, and placental barrier excretes exogenous compounds out of the cells, which thus changes the distribution of the drugs in local tissues. Accordingly, regulating the activity of the P-gp on these tissues may influence the targeted distribution of drugs. The drug entering systemic circulation is excreted out of the cells as original form or its metabolites, which can be accelerated by the P-gp distributed on these tissues. Otherwise, excretion will be put off if the P-gp activity is inhibited, and the blood concentration will be improved [43]. The drug in blood can be reversibly bound with the plasma protein partly, and only the unbound drug can pass through the biomembrane, take effect, and be eliminated. As drugs can be saturated when bound with plasma protein and different drugs have different protein binding rates, they can be competitive at the site of protein binding when two or more drugs are administrated simultaneously, which makes the weakly bound drug free type whose activity and toxicity are thus increased [44]. In conclusion, enzymes, transport proteins, and plasma protein binding can mediate drug interaction in ADME.

### 3.2. Significance of the Research on Pharmacokinetic Compatibility Mechanism of Traditional Chinese Medicine Prescription Based on* In Vivo* Drug Interaction of Main Efficacious Components

#### 3.2.1. Studies on Pharmacokinetic Compatibility Mechanism Based on* In Vivo* Drug Interaction Help to Illustrate Compatibility Principle from the Essence Leading to the Change of Efficacious Components* In Vivo*

Theoretically, drug interaction among herbal ingredients in formulae compatibility may happen at chemical, pharmacokinetic, and pharmacological levels. But its model can generally be classified into two types: one is to change the* in vitro *compositions of active ingredients affecting internal environment of organism in quality and quantity; and the other is to produce* in vivo *cooperative or antagonistic interaction in segment of pharmacokinetics and pharmacological effects. As far as pharmacokinetics compatibility is concerned, drug interaction can happen in the course of ADME and may be manifested by affecting bioavailability of ingredients, changing distribution characteristic, regulating compositions of* in vivo* pharmacodynamic substances, and so on, finally leading to the changes of pharmacological effect. Therefore, studying the pharmacokinetics compatibility is an important way and method for illustrating the mechanism underlying formulae compatibility [45]. However, if these studies are only descriptive and limited to the study of influence on* in vivo* dynamic process of certain ingredients, they would only provide reasonable explanation for formulae compatibility principle from phenomena. It would be helpful to find the principle from the essence leading to the change of effective substances* in vivo* by deeply exploring the transport ways, metabolic pathways and their underlying mechanisms, and the interaction of main efficacious components of compatible drugs in the course of ADME.

#### 3.2.2. Studies on Compatibility Mechanism of Main Efficacious Components Can Simplify the Research on Formulae Pharmacokinetic Compatible Principle

The complication of the formulae composition makes it difficult to study pharmacokinetic compatibility, and it is almost impossible to study all the interactions among all ingredients in a formula. So it is very meaningful to establish a scientific selection and evaluation technology system for understanding the compatible principle ofmain efficacious components and then predict and deduce formulae pharmacokinetic compatibility. Using this strategy, the classical TCM formula should be selected and the interaction of main efficacious components of compatible drugs should be studied with emphasis from possible segments where pharmacokinetic compatibility occurs, including transporters, enzymes, and binding with plasma proteins, which would simplify the research on formula pharmacokinetic compatibility.

#### 3.2.3. Studies on Pharmacokinetic Compatibility Mechanism of Main Efficacious Components Can Provide Guidance for Clinical Application and Efficacious Components Compatibility

If the principle of pharmacokinetic interaction mechanism of formulae compatibility is revealed, it helps to dissect the mechanism of prescription in treating diseases. Using the principles, we could also give the herbs certain nature-like property, taste, and meridian tropism of the herb; combined with the mechanism of herbal pharmacodynamics, we would realize accurate scientific compatibility and overcome the experiential difference of traditional compatibility [43]. Compatibility of herbal efficacious ingredients is becoming a new model for studying modern formulae compatibility, and revealing the essence of compatibility principle at metabolic level is of great importance in guiding efficacious components compatibility.

### 3.3. Suggestion on Pharmacokinetic Compatibility Mechanism of Traditional Chinese Medicine Prescription Based on* In Vivo* Drug Interaction of Main Efficacious Components

After the classic prescription is selected, we should identify main efficacious ingredients and find the potential substrates of metabolic enzymes and transport proteins firstly then study the inhibitory or induction effects of the main efficacious ingredients on the metabolic enzymes and transport proteins. Combined with their metabolic fate and pharmacokinetic behavior, we can find out whether there is drug interaction mediated by metabolic enzymes, transport proteins, or plasma protein binding, so as to illustrate the compatibility mechanism of traditional Chinese medicine prescription. Considering the influence of accompanying substances, the research on pharmacokinetic compatibility mechanism of traditional Chinese medicine prescription based on* in vivo* drug interaction of main efficacious components should be performed at three levels: active ingredients, effective components, and decoction pieces.

Take SGD as an example. Based on the literatures, there is synergistic effect when peony is administrated combined with licorice. The* in vitro* drug interaction is weak in the aspect of the quality and quantity of the chemical ingredients; that is, drug release and new ingredients increase a little. For the* in vivo* drug interaction, on the one hand, synergistic effect is caused by synergistic biological effect; on the other hand, absorption is increased, distribution changed, or metabolism and excretion inhibited. However, there is lack of studies on the essence of these efficacious substances'* in vivo* changes; that is, whether it is because that there exists* in vivo* drug interaction in ADME process of active ingredients that leads to the improvement of bioavailability. Until now, there have been no reports about this yet. Therefore it is necessary to study the drug interaction mediated by metabolic enzymes, transport proteins, or plasma protein binding, so as to clarify the pharmacokinetic compatibility mechanism of SGD.

## 4. Research Design

Based on the literature studies of SGD, albiflorin, paeoniflorin, glycyrrhizic acid, glycyrrhetinic acid, liquiritin and Glycyrrhiza polysaccharide are selected to be the main objective active ingredients. The mechanism of improved bioavailability of main efficacious components would be studied from the following aspects. First, find the transport proteins by studying the effect of transport protein inhibitors on the absorption of the main active ingredients then study the effect on the transport of the specific substrates and the expression of mRNA and protein of the assured transport proteins before and after compatibility of peony and licorice, so as to clarify whether there are* in vivo* drug interactions between peony and licorice by inducing or inhibiting transport proteins. Second, find the metabolic enzymes by studying the effect of metabolic enzymes inhibitors on the absorption of the main active ingredients then study the effect on the metabolism of “probe drugs” and the expression of mRNA and protein of the assured metabolic enzymes before and after compatibility of peony and licorice, so as to clarify whether there are* in vivo* drug interactions between peony and licorice by inducing or inhibiting metabolic enzymes. Third, study the effect of intestinal bacteria on the metabolism of the main active ingredients before and after compatibility of peony and licorice, so as to clarify whether there are* in vivo* drug interactions between peony and licorice by affecting intestinal flora. Lastly, study the plasma protein bindings and their dynamics of active ingredients before and after compatibility of peony and licorice, so as to clarify whether there are* in vivo* drug interactions between peony and licorice by competing the site of plasma protein binding. All the studies should be performed at three levels: active ingredients, effective components, and decoction pieces.

**Figure 1:**
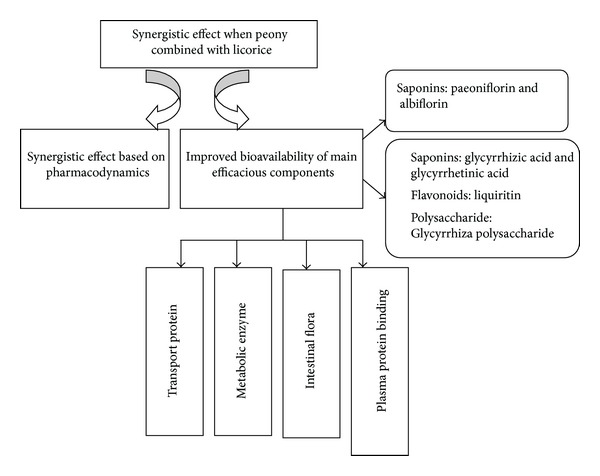
Research design of pharmacokinetics compatibility mechanism of traditional Chinese medicine prescription based on* in vivo* drug interaction of main efficacious components.

Research design is showed in Figure 1.
